# Sample-to-Answer Droplet Magnetofluidic Platform for Point-of-Care Hepatitis C Viral Load Quantitation

**DOI:** 10.1038/s41598-018-28124-3

**Published:** 2018-06-28

**Authors:** Dong Jin Shin, Alexander Y. Trick, Yu-Hsiang Hsieh, David L. Thomas, Tza-Huei Wang

**Affiliations:** 1 Department of Mechanical Engineering, Whiting School of Engineering, The Johns Hopkins University, Baltimore, MD United States; 2 Department of Biomedical Engineering, Whiting School of Engineering, The Johns Hopkins University, Baltimore, MD United States; 3 Department of Emergency Medicine, School of Medicine, The Johns Hopkins University, Baltimore, MD United States; 4 Department of Medicine, School of Medicine, The Johns Hopkins University, Baltimore, MD United States; 5 Infectious Disease Center for Viral Hepatitis, School of Medicine, The Johns Hopkins University, Baltimore, MD United States; 6 Institute for NanoBioTechnology, The Johns Hopkins University, Baltimore, MD United States

## Abstract

Gold standard quantitative nucleic acid tests for diagnosis of viral diseases are currently limited to implementation in laboratories outside of the clinic. An instrument for conducting nucleic acid testing at the point-of-care (POC) that is easily operable by the clinician would reduce the required number of visits to the clinic and improve patient retention for proper treatment. Here we present a droplet magnetofluidic (DM) platform, which leverages functionalized magnetic particles to miniaturize and automate laboratory assays for use in the clinic at the POC. Our novel thermoformed disposable cartridge coupled to a portable multiaxial magnetofluidic instrument enables real-time PCR assays for quantitative and sensitive detection of nucleic acids from crude biosamples. Instead of laborious benchtop sample purification techniques followed by elution and spiking into PCR buffer, the user simply injects the biosample of interest into a cartridge with magnetic particles and loads the cartridge into the instrument. We demonstrate the utility of our platform with hepatitis C virus (HCV) RNA viral load quantitation from blood serum in approximately 1 hour. Clinical serum samples (n = 18) were directly processed on cartridges with no false positives and a limit of detection of 45 IU per 10 µl sample injection.

## Introduction

Chronic hepatitis C virus (HCV) infection is a major public health problem which affects approximately 3.5 million people in the United States^1^. Early stages of HCV are often asymptomatic, so most persons living with HCV are unaware of their infected status, and consequently do not receive the timely care and treatment needed to prevent severe HCV-associated complications including cirrhosis and hepatocellular carcinoma (HCC)^2^. For the majority of population outside the baby boomer cohort, current U.S. guidelines recommend limiting HCV screening to high-risk individuals, a strategy which neglects up to 49–75% of infected individuals who are unaware of their infection^2,3^. Such an approach will result in approximately 1.76 million individuals in the US developing cirrhosis and 400,000 individuals developing HCC in the next 40–50 years^4^.

Missed opportunities in the HCV care pathway point to technical bottlenecks. The Centers for Disease Control and Prevention (CDC) recommends a diagnostic algorithm for HCV that first screens patients with an HCV antibody test, which requires a second RNA-based confirmatory test once a reactive result is obtained. A recent assessment of hepatitis C treatment cascade has shown that only about 60% of all screened individuals are retained for RNA confirmatory testing^5^, representing a major loss along the hepatitis C cascade of care framework. To improve opportunities for earlier identification and linkage to care for the broader population, technical innovations are required to create an HCV test that can be completed with definitive results during the patient visit^6^. Such a point-of-care (POC) test requires integrating patient sample preparation and nucleic acid amplification test (NAAT) into a single disposable device. Traditional microfluidic approaches utilizing microchannel-based architecture have struggled to offer a scalable solution to assay integration, due to their dependence on complex device fabrication and instrumentation^7–9^.

Recent innovation in assay miniaturization and integration via droplet magnetofluidics (DM) create an opportunity to surmount these technical challenges^10,11^. DM technology replaces bulk fluid transport with magnetic particle manipulation through static discrete microliter droplets, enabling integration of bioassays without the need for complex fluidic cartridges and supporting instrumentation. Magnetic particles are capable of transporting, mixing and separating liquid reagents on small devices ranging from soft lithography-based devices^9,12,13^, glass-based substrates^14–16^ to thermoplastic cartridges^17^, facilitating a novel approach to miniaturize and integrate laboratory-bound processes such as nucleic acid extraction on a single device.

To date, assay integration using DM was confined to designs utilizing a single planar substrate for particle manipulation and reagent confinement. Planar substrates offer a favorable interface for particle actuation, whether performed manually by hand^9,12,15^, electromechanically-actuated^16,17^ or electromagnetic^13,14^ approaches. However, planar design also presents a major challenge for biochemical assays. Specifically, the limited capacity for thermal isolation makes processes such as quantitative PCR and melting curve analysis difficult to implement in a precise and scalable manner. In the case of HCV, quantification of viral RNA in blood is an important factor in the evaluation of a patient’s response to treatment and genotype is a strong predictor for HCV prognosis and responsiveness to treatment^18^. Implementation of a quantitative reverse-transcription PCR (qRT-PCR) assay requires thermal cycling with precise control and sufficient heating/cooling rates to complete within a practical time frame. Achieving these goals requires a decoupling of DM manipulation from reagent confinement, in order to combine the benefits of a dedicated thermal cycling module and a DM-enabled sample preparation module.

In this work, we present a sample-to-answer qRT-PCR system for HCV diagnosis, utilizing DM in thermoformed plastic cartridges. The extruded well design of the cartridge enables precise rapid thermal control of reagents at suitable ranges for PCR thermal cycling and melt curve analysis. DM sample processing enables the necessary purification of nucleic acid targets from clinical samples to obtain quantitative and consistent assay results. The DM-PCR platform demonstrates high analytical sensitivity with HCV RNA positive samples ranging from 6,000,000 IU/mL down to approximately 45 IU in serum (equivalent to 4,500 IU/mL) and shows high correlation with laboratory-based viral quantification and benchtop PCR. Patients experiencing chronic infection typically have viral counts on the order of 10^5^–10^7^ IU/ml and desired responses to treatment are characterized by orders of magnitude decreases in the viral load^19,20^. Therefore, the sensitivity of our platform is suitable for detecting most cases of chronic HCV infection with sufficient capacity for viral load quantitation to evaluate patient responses to treatment.

## Results

### Droplet magnetofluidic PCR (DM-PCR) platform overview

**Figure 1:**
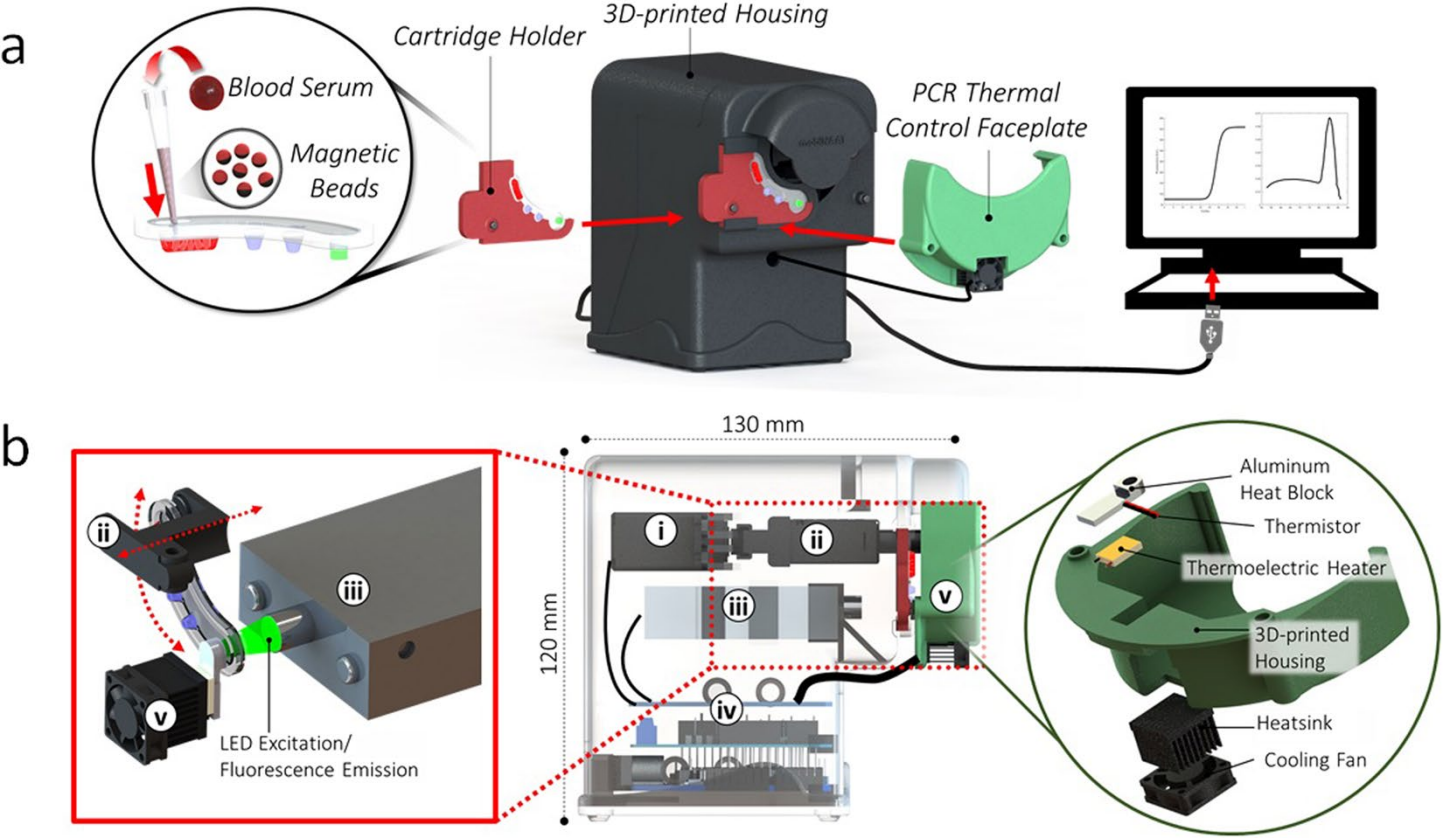
Device operation and instrumentation (**a**) the complete user-operation steps are illustrated here from left to right including injection of serum with magnetic particles into the cartridge, cartridge mounting in a holder with insertion into the instrument, assembly of the faceplate onto the cartridge for thermal cycling, and USB readout of PCR results. (**b**) A profile view of the instrument with ghosted housing reveals internal components including (i) rotational servo and (ii) linear servo for magnetic particle transferal between cartridge wells and in/out of reagent droplets respectively, (iii) confocal epifluorescence detector for PCR fluorescence acquisition, (iv) microcontroller electronics for integrated control of magnetic manipulation, algorithmic heating, triggering fluorescence detection, and serial communication for data storage and analysis, (v) PCR thermal control faceplate with a fan-cooled heatsinked thermoelectric heating element attached to an aluminum heat block that fits over the cartridge PCR well with an embedded thermistor probe for monitoring temperature.

Two of the major technical bottlenecks in the implementation of qRT-PCR diagnostic tests include (1) laborious sample processing steps for nucleic acid purification, and (2) the need for trained laboratory personnel to operate instruments for complex biological assays. The DM-PCR instrument overcomes both issues by automating sample processing on magnetic particles within a single cartridge to couple nucleic acid purification directly with temperature control for enzymatic amplification and fluorescence detection for quantification and melt analysis. Operation requires only a single injection of biosample such as blood serum mixed with magnetic particles into a cartridge followed by insertion into our instrument to initiate an assay (Fig. 1a).

DM manipulation of the injected sample is facilitated by a dual-axis (Z-θ) actuator, which drives mechanical displacement of two permanent magnets using microcontroller-driven RC servomotors. Vertical displacement (Z) is achieved via linear RC actuation, while radial displacement (θ) is achieved on a conventional rotary RC servo. Thermal control is implemented by a faceplate that mounts directly onto the well of the cartridge containing the PCR buffer. A thermoelectric (TE) element-driven miniature heat block heats and cools the PCR well with temperature feedback from a thermistor probe embedded directly in the heat block. Optical detection utilizes a miniaturized confocal fluorescence detector at a fixed distance from the PCR well. Transparency of the top layer of the assay cartridge enables sensitive detection of fluorophores over a dynamic range calibrated to 0.5–50 nM fluorescein (Fig. S1). The three modules are controlled by an Arduino microcontroller and interfaces via serial communication to a Java-based control software (Fig. S2). See Fig. 1b for a schematic of all internal components of the instrument.

### Magnetic actuation

**Figure 2:**
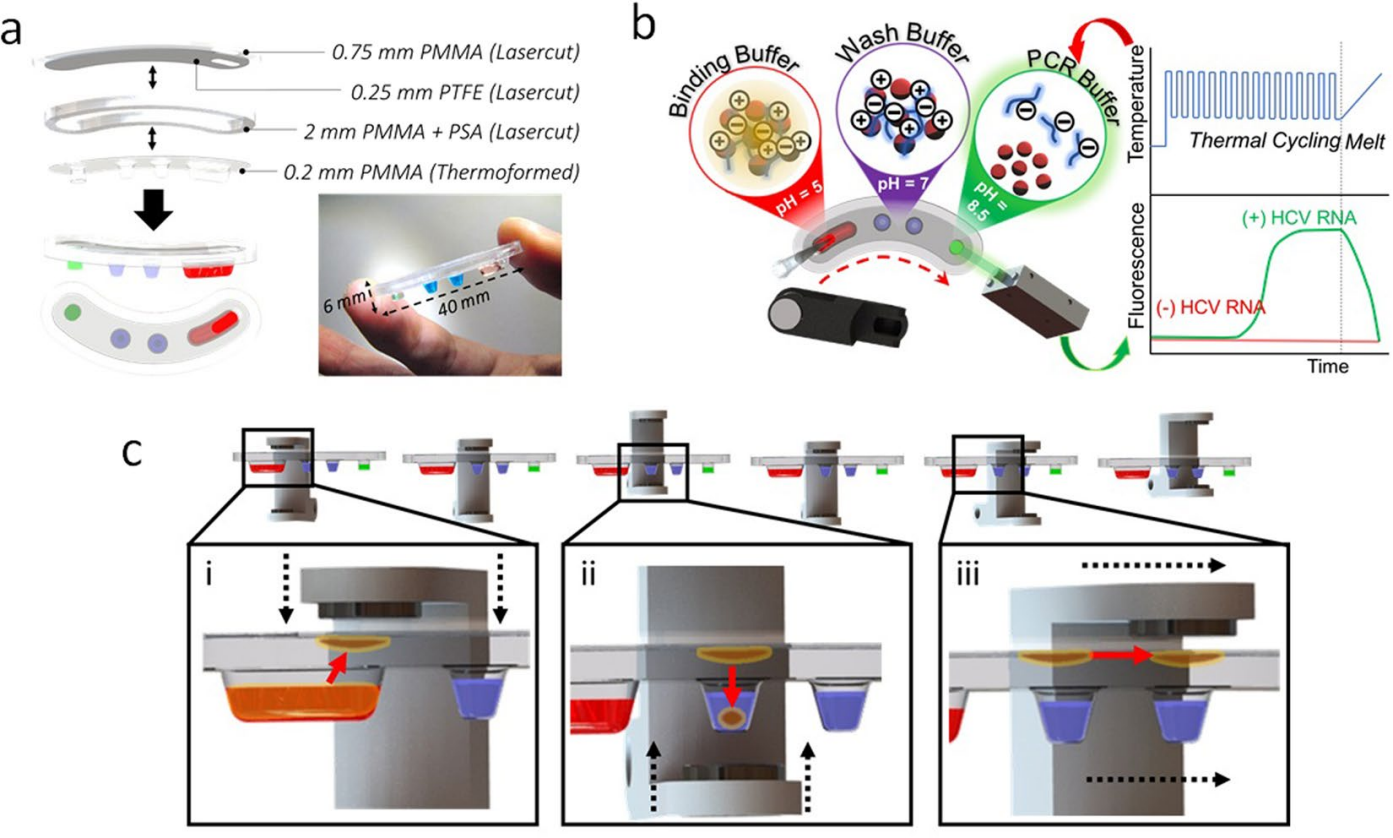
Cartridge Design. (**a**) Cartridges are fabricated with three parts: a lasercut top layer of poly(methyl methacrylate) (PMMA) with a coating of poly(tetrafluoroethylene) (PTFE) tape for smooth particle plug transfer between wells, a middle laser cut layer of PMMA as a spacer for containing silicone oil in the transition region between wells, and a bottom vacuum-formed PMMA layer with extruded wells for containing reagents. Pressure sensitive adhesives (PSA) applied to both surfaces of the middle spacer generates a leak-proof seal upon cartridge assembly. (**b**) Overview of nucleic acid purification and PCR assay droplet reagents with their arrangement within cartridge wells. Samples of interest are injected with magnetic particles into the first well containing 40 µl of a binding buffer (pH = 5) through an opening in the top of the cartridge. At this low pH, the polyhistidine coating of the magnetic particles is positively-charged, which allows binding to negatively-charged nucleic acids by electrostatic forces. Transfer of magnetic particles through two wash buffers (pH = 7) purifies the captured nucleic acids via desorption of unbound cellular debris or proteins that may inhibit the PCR reaction. Final transfer into the PCR solution (pH = 8.5) neutralizes the charge of the magnetic particle surface allowing for elution of nucleic acids for thermal cycling and subsequent melt analysis coupled with fluorescence detection. (**c**) Particle transfer actuation is accomplished by (i) descent of the upper magnet to draw particle plug into oil for collection at the PTFE surface, (ii) ascent of the lower magnet to introduce particle plug into reagent droplets, and (iii) magnet rotation for particle transfer between wells.

Previous iterations of DM devices describe an essentially 1-dimensional system, where all reagents and passive barrier structures separating them are located on a single plane. Such a system only requires actuation along a single dimension (e.g. X, Y or θ) to perform magnetic particle transport across all reagents in the system. In the thermoformed magnetofluidic cartridge described here, extruded wells and an immiscible oil layer serve to separate and constrain reagents within discrete wells. In order to extract and transport particles, the ability to manipulate particles in and out of the extruded wells is required. The Z-θ manipulator facilitates automation of this process. Extension and retraction along the Z-axis draws magnetic particles in and out of the wells, while rotation (θ) provides transfer between wells. In the revised magnetofluidic manipulation scheme, as illustrated in Fig. 2c, the surface tension of the oil-water interface serves to sieve off excess immiscible solution encapsulating the particles for clean transfer between wells without compromising droplet integrity or mixing reagents (Fig. S3).

### Cartridge design

The DM-PCR cartridge consists of 3 layers: a thermoformed reagent layer, a spacer, and a hydrophobic transport layer (Fig. 2a). The reagent layer utilizes a thin thermoplastic sheet to generate thin-walled extruded wells akin to conventional PCR tubes, facilitating rapid thermal control necessary for PCR-based assays. The spacer layer confers volume to the cartridge such that an immiscible layer of silicone oil insulates all reagents from the surrounding air and each other. The hydrophobic transport layer employs a poly(tetrafluoroethylene) (PTFE) coating to provide a common plane on which magnetic particles may slide with minimal friction or sample loss upon contact. Pre-loaded reagents and a leakproof seal facilitated by pressure-sensitive adhesive on either side of the spacer layer results in a compact, disposable product with “out-of-the-box” functionality.

The cartridge demonstrated in this work contains four wells preloaded with droplets of a binding buffer, two wash buffers, and a PCR reaction mix. The assay, as illustrated in Fig. 2b, begins with injection of a sample (e.g. blood serum) mixed with pH-responsive magnetic particles into the first well through a port in the cartridge. This first well contains the acidic binding buffer, which induces a positive charge on the polyhistidine coating present on the particle surface. Electrostatic forces then cause binding between the magnetic particles and negatively-charged nucleic acids in solution. Blood-derived biosamples may contain numerous factors that are inhibitory to PCR such as IgG, hemoglobin, and lactoferrin^21^. Transfer of the particles through the two wash buffer wells ensures that inhibitory components from the sample are desorbed from the particles while the pH maintains the positive surface charge on the particles for subsequent transfer of the captured nucleic acids into the PCR solution. PCR solutions are inherently basic (pH 8.5), which neutralizes the magnetic particle surface charge for elution of sample nucleic acids. The magnetic particles are then transferred out of the well followed by PCR thermal cycling and melt curve analysis.

### Temperature control in thermoformed assay cartridge

Thermal cycling and predictable heat control presents various technical challenges for integrated implementation of PCR with assay preparation on a single cartridge. All elements of the assay must be accessible in a continuous path within the cartridge while providing a method for directing localized heating to the isolated PCR volume. Thermal cycling is a nontrivial operation for planar magnetofluidic devices due to geometrical constraints imposed by their planar design. Using thin, thermally conductive substrates such as glass coverslips can achieve smaller thermal gradient across the device substrate and will enable thermal cycling of PCR reagents on device^15^, although the substantial thermal mass of all reagents on the substrate results in higher temperature ramp times and an extended reaction time compared to a conventional PCR system. In this work, we addressed these challenges with thermoformed extruded wells (Fig. 2a).

Thermoforming serves as a scalable fabrication method to easily generate thin-walled extruded wells for physical isolation of the PCR reagent. Extrusion of the wells facilitates an increase in the surface area of the heating element in direct contact with the incubation region of the cartridge. The materials chosen for the cartridge needed to be amenable to thermoforming, transmit light for fluorescence detection, and avoid inhibitory interactions with PCR components. Poly(methyl methacrylate), also known as acrylic, was chosen for optical transparency and compatibility with both laser-cutting and thermoforming techniques. Acrylic has been shown to inhibit PCR and prevented sensitive detection of nucleic acids in our cartridge^22^. This issue was circumvented by passivating the wells with silicone oil prior to loading reagents.

**Figure 3:**
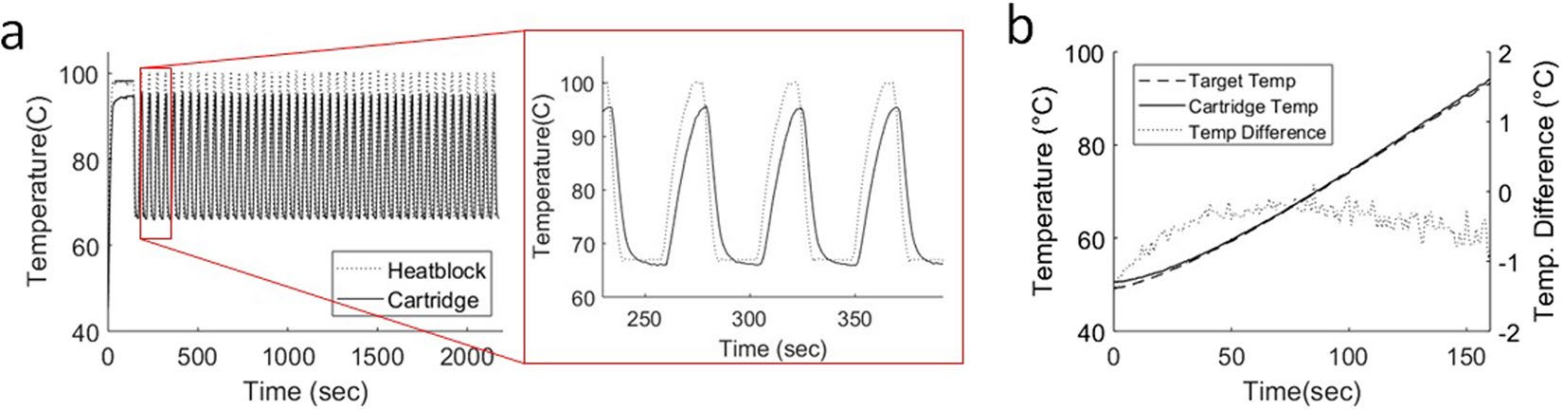
PCR temperature consistency. (**a**) Thermal cycling profile as measured by thermistors within the heat block (dashed line) and embedded within a test cartridge PCR well (solid line). PCR assays demonstrated here used a 2-minute hot-start at 95 °C followed by 45 cycles of 20 seconds at 65 °C anneal and 5 seconds at 95 °C denature. (**b**) Temperature ramp profile demonstrating close agreement between the targeted temperature with the actual measured temperature in the cartridge with error measuring <1 °C throughout the relevant range of temperatures for melt analysis of amplified nucleic acid products.

**Figure 4:**
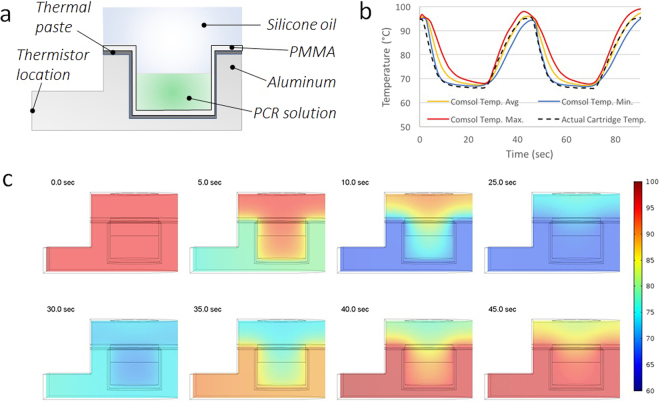
PCR Thermal Cycling Characterization. (**a**) Cross-sectional schematic of the aluminum heat block with inserted PCR well of thermoformed cartridge. Thermally conductive paste was used to ensure complete contact and heat transfer between the heat block and cartridge. (**b**) Comparison of maximum, average, and minimum temperature of the PCR solution tracked during finite-element COMSOL simulation versus actual temperature measured by a separate thermistor directly within the cartridge well during thermal cycling. (**c**) Temperature heat-map snapshots from cross-section of the finite-element simulation. The top row follows cooling from a uniform temperature of 95 °C while the bottom row shows the heating back to 95 °C. Uniform temperature distribution within ~1 °C of the target annealing and denaturation temperatures is evident throughout the entire PCR solution delineated by the rectangle at the bottom of the well at the end of each heating and cooling phase.

To transport heat to the cartridge, an aluminum heat block was machined to encompass the PCR well. The low-profile design of the heat block both reduces thermal mass for rapid heating by the attached thermoelectric element and permits sufficient range of movement by the magnet arm for magnetic sample exchange with the PCR well. Because the cartridge is completely enclosed during thermal cycling, monitoring the temperature of the PCR solution was done indirectly with a thermistor embedded into the heat block adjacent to the well. The temperature correspondence between the thermistor reading and PCR solution temperature was found to follow a linear trend by embedding a second thermistor within the PCR well of a test cartridge (Fig. S4). Temperature targets were calibrated accordingly to follow this trend and thermal cycling accuracy and consistency was evaluated using both an embedded thermistor within the cartridge and a finite-element heat transfer simulation (Figs 3a and 4). Furthermore, a thermal ramp calibration test between the heat block and PCR well demonstrated the instrument’s ability to generate a linear thermal ramp over the relevant range of a typical melt curve analysis (Fig. 3b).

After calibrating cartridge temperature to heat block temperature, thermal cycling for 45 cycles was employed using a PID algorithm to control heating with a 5 second denature and 20 second anneal using 95 °C and 65 °C target temperatures respectively to match temperatures for our HCV assay. The PCR well thermistor measured consistent average temperatures of 95.0 ± 0.5 °C and 66.2 ± 0.1 °C at the end of each cycling step (Fig. 3a). Subsequent temperature ramping from 50 °C to 95 °C over the course of ~3 minutes using the same calibration maintained accuracy to the target temperature within <1 °C (Fig. 3b). The finite-element simulation of the cartridge given the same temperature parameters for thermal cycling closely agreed with the results from our embedded thermistors and validated that a uniform temperature profile was established at the end of both annealing and cooling (Fig. 4b,c).

### Cartridge PCR and melt analysis evaluation

**Figure 5:**
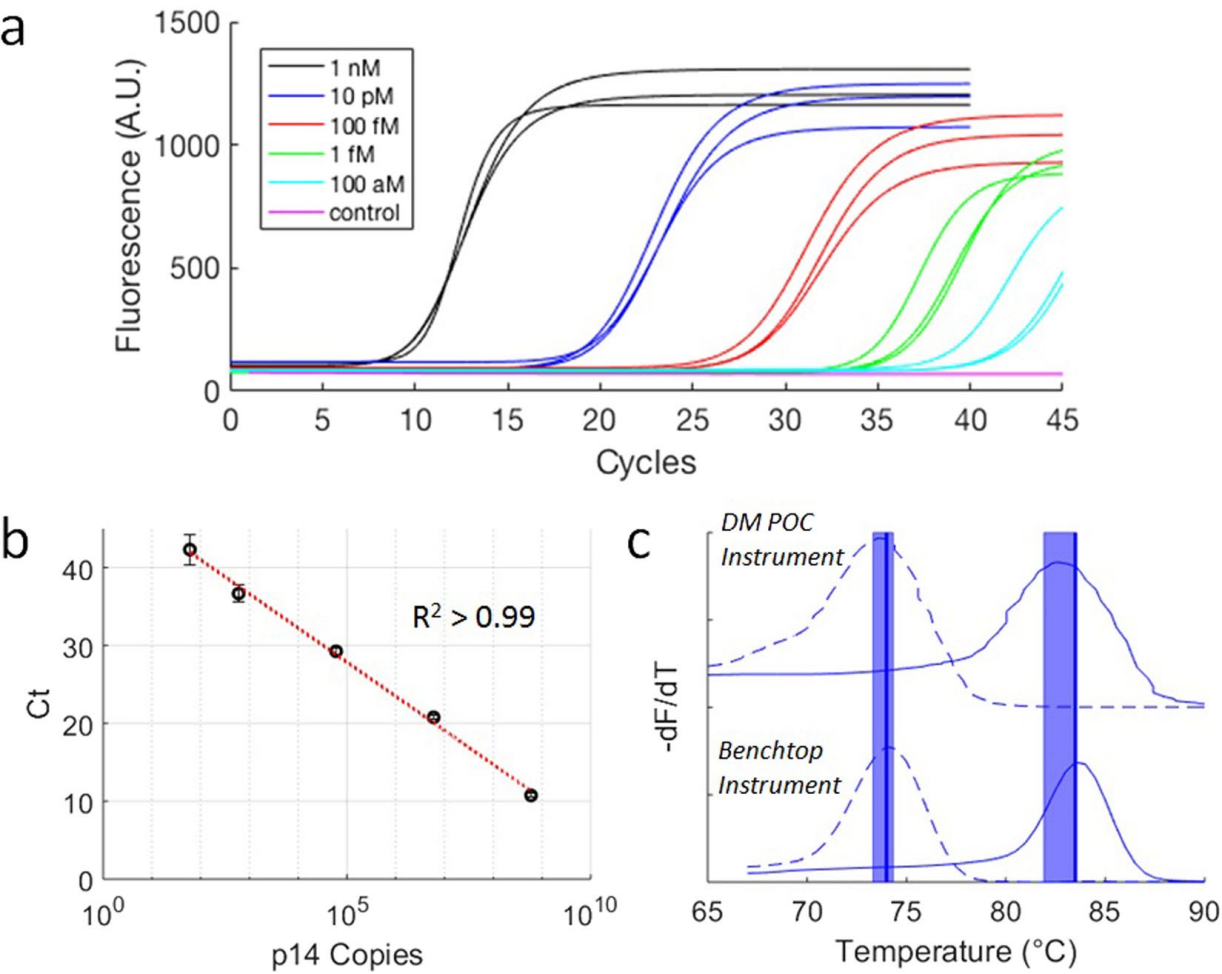
Cartridge PCR and melt performance. (**a**) Real-time fluorescence of PCR thermal cycling on cartridge using 10 µl PCR solutions directly spiked with dilutions of synthetic p14(ARF) oligonucleotide targets ranging from 1 nM to 100 aM in concentration (n = 3 for each dilution). No specific amplification was detected with a negative control (pink). (**b**) Standard curve generated from the cycle threshold (Ct) values calculated from the PCR in (**a**). (**c**) Comparison of p14(ARF) amplicon melt curves generated by our instrument (top) with those generated by a commercial benchtop thermocycler (bottom). The dashed lines indicate the low melt temperature curve of a shorter nonspecific product that is amplified when a PCR solution is left at room temperature for several hours, while the solid lines are melt curves of the desired amplicon products produced when the target is present in the PCR solution. The dark vertical lines represent the benchtop thermal cycler melt temperatures (Tm = 74 °C and 83.5 °C) which had a 0.5 °C resolution and were consistent between runs. The broader vertical bars denote one standard deviation from the average melt temperatures detected with the cartridges (Tm = 73.8 ± 0.5 °C and 82.7 ± 0.8 °C).

PCR performance on the cartridge was characterized using synthetic human p14(ARF) oligonucleotide targets and primers (Table S1). The PCR conditions were first evaluated with serial dilutions of target on a standard benchtop thermal cycler (Fig. S5) before testing PCR with directly spiked targets in the cartridge system. Serial dilutions of p14(ARF) targets were tested in triplicate on our cartridge system with 1 µl target inputs per 10 µl reaction volume with concentrations ranging from 1 nM to 100 aM (Fig. 5a). The p14(ARF) targets were detectable across the full range of concentrations, including at 100 aM corresponding to approximately 60 copies per reaction (Fig. 5b). A strong linear relationship (R^2^ > 0.99) between algorithmically determined cycle threshold (Ct) and log[p14(ARF) copies] demonstrates consistency in the implementation of PCR thermal cycling on cartridge. Melt temperature analysis on cartridge and benchtop showed close agreement within <1 °C (Fig. 5c). Nonspecific products as a result of undesired polymerase activity on oligonucleotide primers in PCR solution were easily differentiable by melt temperature from specific amplicons. This ability to distinguish products by melt peaks provides a method for screening false positives and potential for multiplexed assays that is unavailable with standard protocols for isothermal techniques like LAMP typically investigated for POC instrumentation^23^.

### Using the DM-PCR platform to process clinical specimens

We evaluated the HCV RNA assay using serum samples obtained from two groups of individuals defined using two commercial ‘gold standard’ HCV assays. The first group was composed of patients with chronic HCV infection in whom HCV RNA was detected by NAAT. The second group contained individuals who had cleared HCV infections as indicated by detection of HCV antibodies in serum, but no detectable HCV RNA by NAAT. We designed a primer pair to enable specific, pan-genotypic amplification of 5′ untranslated region (UTR) in the HCV genomic RNA. Evaluation of our primer set using a benchtop processed clinical sample on a standard thermal cycling instrument demonstrated linearity for target concentrations of 48–6000 IU per reaction (Fig. S6). Evaluation of the primer set with all HCV-positive serum samples demonstrated a limit of detection of 12 IU of RNA and successful amplification of genotypes 1a, 2b, and 3a (Table S2). Quantification of all HCV-positive serum samples by Ct (Fig. S7) yielded a very high degree of correlation with clinically reported values for HCV RNA quantities (R^2^ = 0.97).

**Figure 6:**
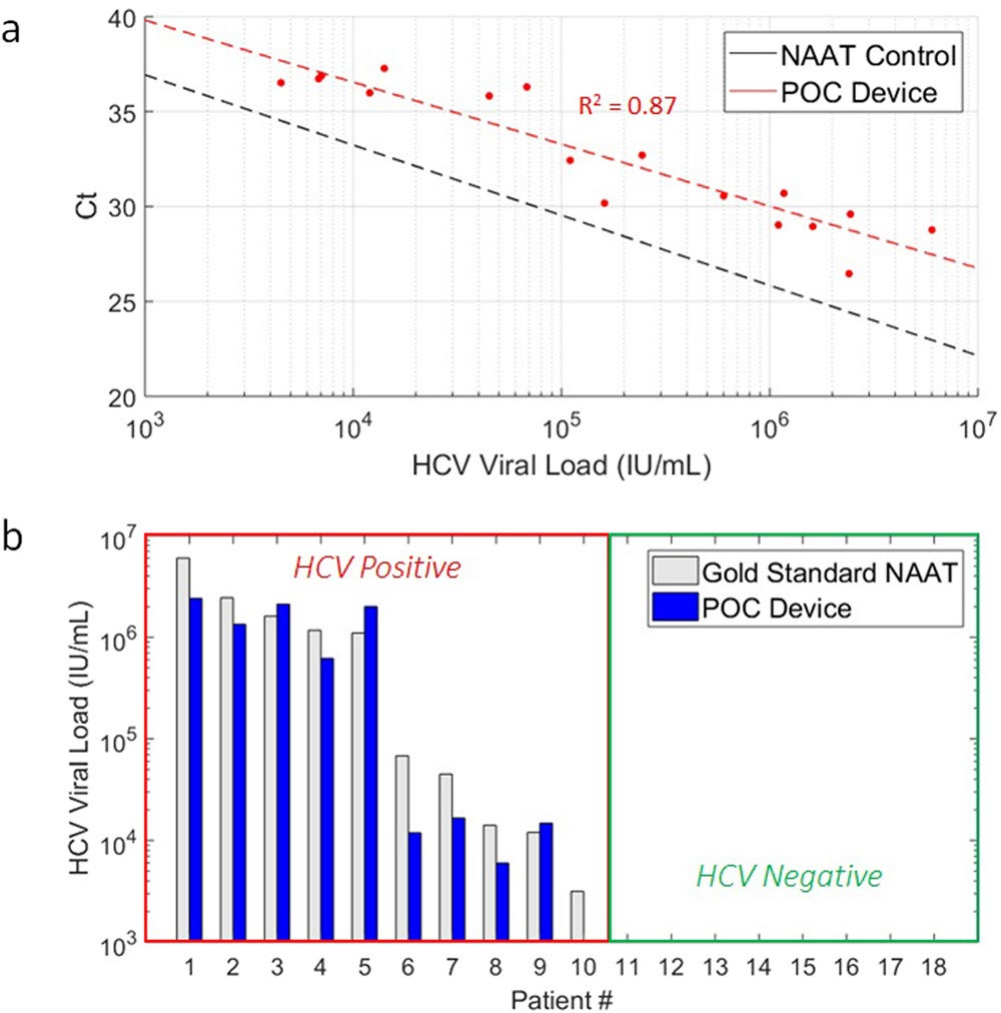
Clinical serum viral load evaluation. (**a**) Standard curve for threshold cycles versus HCV RNA input generated from cartridge (red) compared to standard curve obtained from NAAT control conducted on a benchtop thermocycler (Fig. S7). Nine HCV-infected clinical blood serum samples (undiluted and 10% serum in water, n = 18) were injected into cartridges in 10 µl aliquots. The limit of detection was found to be approximately 45 IU of HCV RNA. (**b**) Measurement of HCV RNA from 10 µl undiluted serum samples using DM-PCR assay cartridges. Estimates of HCV IU (blue bars) were calculated by substituting Ct values from each cartridge PCR test into the fitted relationship represented by the dotted red line in (**a**). The actual values (gray bars) were based on HCV IU concentrations for each patient reported by clinical laboratory processing. Only patient 10 with the lowest viral load (3,160 IU/ml) went undetected out of all positive samples (n = 10). No false positives were detected through cartridge processing of the cleared HCV negative patient samples (n = 8).

After evaluation using commercial ‘gold standard’ HCV assays, each serum sample was tested on our platform by injection into pre-loaded cartridges with magnetic particles and subjected to automated nucleic acid purification, RT-PCR, and melt curve analysis. Results from cartridge device using 10 µl aliquots of HCV-positive clinical samples with viral loads in the range 10^3^–10^7^ IU/mL shows a clear inverse relationship (R^2^ = 0.87) between viral load and Ct (Fig. 6a) with a 2.8–3.4 cycle increase compared to the benchtop standard in detection of HCV IU in the range of 10^1^–10^5^. The limit of detection on cartridges was demonstrated at 45 HCV IU per injection (Fig. 6a). A standard curve generated from this data provides a method for estimation of viral load within an order of magnitude (Fig. 6b).

In a conventional PCR assay utilizing double-stranded DNA indicators such as SYBR Green, melt curve analysis is often used to test for product specificity. Melt curve analysis is a post-amplification analysis technique utilizing double-stranded DNA indicators to determine the melting temperature (Tm) of nucleic acids. Dissociation of nucleic acids can be monitored over time by measuring fluorescence while subjecting the sample to thermal ramp over time. This signature is unique to any given nucleic acid sequence due to the thermodynamic differences attributed to the sequence length and base composition. In our test of clinical specimens, negative serum samples displayed no amplification or late amplification of non-specific primer-dimer products as indicated by a lower melt temperature than seen in the positive samples. Melt curve analysis on cartridge measured a melt temperature of 85.9 ± 0.8 °C for positive samples, which corresponds well with the benchtop melt temperature range of 86.5–87.0 °C. Similarly, nonspecific amplicons from our primer set produced a melt temperature of 79.9 ± 0.4 °C on our platform compared to 79.5–80 °C melt temperatures on the benchtop instrument (Fig. S7b).

## Discussion and Conclusion

DM platforms have previously been limited to isothermal or off-chip thermal cycling due to planar designs that restrict localized and rapid heat transfer. By expanding magnetic manipulation of samples beyond a single plane with a Z-θ magnetic manipulator, we demonstrate integration of sample processing and PCR thermal cycling within a single cartridge. Sequestration of digitized reagent droplets was accomplished with thermoformed acrylic extruded wells and silicone oil as an immiscible transfer medium. Assay operation is simplified to a single injection into a cartridge followed by insertion into our instrument. The ease of operation and highly scalable thermoforming manufacturing process makes this platform suitable for translating PCR NAATs to the POC. Enabling PCR at the POC provides significant advantages to current trends in the development of POC technology toward isothermal amplification NAATs due to higher sensitivity, potential for faster assays, multiplexing capabilities, quantification, and high-resolution melt analysis.

HCV RNA detection is critical for evaluation of a patient’s current state of infection and for monitoring treatment success. Whereas precise and sensitive quantification was once necessary to make treatment decisions, now treatment is indicated for all with detectable HCV RNA and considered successful for all for whom no HCV RNA is detected 12 or more weeks after treatment cessation^24^. Indeed, we and others have demonstrated considerable loss of patient retention between visits at which screening and confirmation of infection are done^25,26^. Clearly, one of the major limitations in the overall effectiveness of treatment is the inability to deliver complete care at the point of service. In particular, a lack of direct accessibility to diagnostic instrumentation prevents concurrent evaluation of HCV viral load and clinical consultation within a single visit. Use of our cartridge-based system could simplify diagnosis and viral load monitoring to a single visit to reduce attrition due to loss of follow-up as well as the enormous psychological harm that occurs when a patient is told infection is possible but not yet confirmed. Confirmation at the point of care also allows for immediate prescription of treatments to the patient or appropriate adjustments based on disease progression.

Although the dynamic range demonstrated on our device does not match the entire range of viral loads seen clinically, its performance is more than sufficient for screening a large majority of patients with chronic HCV infections—a test that is not possible in clinics with limited laboratory and financial resources given costs of $30 to $200 per test^27^. Typical HCV RNA levels in blood of chronically infected patients occur between 10^5^–10^7^ IU/ml, and maintain consistent levels within individuals if gone untreated^28^. Our cartridge has a demonstrated sensitivity of 45 IU per sample, corresponding to approximately 4,500 IU/ml, which adequately meets the threshold needed for diagnosis and identification of significant changes in a patient’s viral load. In one analysis of multiple patients enrolling in a clinical trial, 97.2% had viral loads greater than 4,500 IU/ml^29^. Decreases in the viral load by 2 orders of magnitude, as well as cut-off ranges between 500,000 and 4,000,000 IU/mL have been studied as effective benchmarks for prediction or evaluation of a patient’s response to various drug treatments^30–32^. Our system has a demonstrated viral load estimation from samples with HCV RNA concentrations spanning 5 orders of magnitude with an estimated error <1 log(HCV RNA), highlighting our device’s potential application in monitoring HCV treatment efficacy.

While the data obtained using the magnetofluidic device is mostly in agreement with the viral load determined using the standard of care assay, some differences in performance can be observed. Importantly, the device-generated data exhibits a greater spread and is currently unable to detect target inputs at 31.6 IU and below. While the spread of observed data may be substantially mitigated in a production-ready device beyond the prototype demonstrated in this work, sensitivity remains a challenge. Retrieving viral RNA templates from serum using a solid-phase extraction technology presents part of the issue, and may only be addressed by increasing the sample volume by another order of magnitude from the current input of 10 µl. It is envisaged that future iterations of this technology could utilize samples collected using finger-stick collection tubes, which typically handle volumes in the range of 100–250 µl. Considering the typical volume of serum sample utilized in commercial HCV RNA tests to be around 500 µl, our approach demonstrates a substantial reduction in input sample volume while retaining assay sensitivity over a broad range of clinically applicable HCV RNA levels in blood.

Our assay was designed for pan-genotypic amplification of all HCV viral strains with demonstrated success in detection of genotypes 1a, 2b, and 3a. This goal is consistent with the recent development of pan-genotypic treatments. Development of drugs like Vosevi® (Gilead Sciences, Inc.) and Mavyret™ (AbbVie Inc.) provides pan-genotypic treatment options for naïve patients. Nonetheless, genotyping HCV infections remains the standard of care^33,34^. Notably, current commercially available tests such as the VERSANT® HCV genotype 2.0 assay (LiPA) (Siemens Healthcare GmbH©) could be adopted into our cartridge system. Primers could also be designed to produce amplicons with varying melt temperatures based on each genotype. Given a standard deviation <1 °C for cartridge melt temperatures of the genotype 1a clinical samples, designing primers for genotype differentiation would require only a few degrees difference to tell them apart using melt analysis. The fluorescence detector used in our platform also has 2-color excitation and emission functionality, which could be leveraged for multiplexing for genotyping or diagnosis of additional RNA viral diseases like HIV. By bringing diagnostics to the POC with our platform, HCV screening could be made more widely available for early detection of HCV before development of liver cirrhosis, enabling use of these pan-genotypic treatments and simplicity of the cartridge assay to the pan-genotypic amplification in this work.

In the tests demonstrated on our cartridges, clinically obtained serum was injected rather than whole blood samples. To extract blood serum from whole blood traditionally requires expensive centrifuges, which presents another roadblock to implementation of assays outside of a typical laboratory environment. Recent advances in POC and lab-on-chip technology have produced simple low-cost methods for blood separation in minutes that use disposable devices leveraging hydrophobic surfaces or hand-powered centrifugation^35,36^. Another barrier to realistic implementation of our cartridges is long-term storage of PCR reagents. Enzymes involved in NAATs are sensitive to degradation and will produce non-specific products if primers are present leading to potentially false-positive results. To circumvent this storage issue, lyophilization of PCR solutions and encapsulation in wax for release upon heating is one successfully demonstrated technique that could easily be adopted into our cartridges^37–39^. All other necessary components besides the PCR solution (binding buffer, wash buffers, magnetic particles) are stable at room temperature for convenient cartridge storage. Pairing our platform with novel methods for plasma separation and reagent storage would remove the need for laboratory entirely, enabling complete sample processing and clinical answer in the same room as the patient.

The potential uses for our magnetofluidic POC PCR platform extend well beyond testing for HCV. The multi-well design of our cartridges allows for carrying out a variety of other assays involving the capture and transport of nucleic acids. In particular, the DNA methylation characteristics of certain genes show promise for use in early cancer detection. Assessment of methylation levels of DNA through bisulfite conversion on magnetic particles has been previously demonstrated in a magnetofluidic system, but required manual extraction of eluted product for PCR on a benchtop system^40^. Using the paradigm of our extruded well cartridge design, the complete procedure for analysis of DNA methylation levels from cell lysis to NAAT could be completely integrated. Outside of healthcare, POC-PCR with magnetofluidics could expedite analysis of crops and livestock in agricultural settings for on-site detection of bacterial contamination or genotyping for identification of genetically-modified strains^41–43^.

## Materials and Methods

### Design and fabrication of portable PCR instrument

The instrument utilizes an Arduino microcontroller (Arduino Uno R3) with two stackable custom printed-circuit boards (PCB) for connection and communication with all electronic components. The Z-θ manipulator was built by coupling the base of a PQ12-R linear servo (Actuonix, Victoria, BC, Canada) to the rotary shaft of HS-485HB rotary servo (Hitec RCD, Poway, CA, USA) using a custom-designed coupler. All custom mechanical components were designed using a computer-assisted drawing (CAD) software (Soldiworks 2015, Dassault Systemes SOLIDWORKS, Waltham, MA, USA) and built using a desktop 3D printer (Formlabs Inc., Somerville, MA, USA).

The heating module consisted of five components as shown in Fig. 1b: an aluminum heating block, a thermoelectric (TE) module, a temperature probe, a heatsink and a fan. The heating block contained a machined well to mate with the cartridge and was designed to maximize contact with the TE module while minimizing thermal mass and vertical profile to enable unimpeded access to the PCR well by the Z-θ manipulator. The TE module was purchased from Custom Thermoelectric (Bishopville, MD). A temperature probe was built using a 14 kΩ NTC thermistor (Semitec USA, Torrance, CA) in a Wheatstone bridge configuration. The thermistor head was embedded into the heating block to monitor temperature. The heatsink and fan were used to dissipate heat on the TE module during the cooling phases of thermal cycling. A PID algorithm was used to control current to the TE module to adjust and maintain temperature based on readings from the thermistor probe.

### PCR and melt fluorescence data analysis

A software control interface was programmed using the open-source Processing IDE. In brief, the control interface utilized serial communication to the Arduino microcontroller to send instructions to the optical, thermal and mechanical modules. Data acquired from optical and thermal modules was sent back to the control interface. An overview of the software interface is described in Fig. S2. Data analysis was carried out with a custom MATLAB script as described in Fig. S8. Fluorescence peak values from each cycle were isolated and fit to a logistic regression curve for Ct determination by the second derivative maximum^44^. Melt temperatures were determined by smoothing the fluorescence signal versus temperature using a Savitzky-Golay filter. The negative first-order derivative (−dF/dT) was calculated for the resulting curve and the peaks were identified as the melt temperature.

### Design and fabrication of thermoplastic cartridge body

The cartridge consists of two functional layers separated by a spacer. The bottom layer consists of extrusions designed to hold assay reagents while providing adequate thermal contact for the PCR reagent. The top layer consists of an inlet for pipette-loaded injection of sample into cartridge, in addition to a smooth PTFE surface to facilitate magnetic particle transport between reagents. Each cartridge component was first designed using CAD software and later developed into either a laser-cutting template or a vacuum mold as appropriate for prototyping.

The cartridge is built using a combination of laser-cutting and thermoforming (Fig. S9). First, a thin (0.2 mm) PMMA film is thermoformed into a multi-well bottom layer using a commercial dental vacuum forming instrument (Meta Dental Corp, Glendale, NY). Afterwards, a thicker (0.75 mm) PMMA sheet is laminated on one surface with PTFE tape and engraved or cut using a CO2 laser cutter (Universal Laser Systems, Inc., Scottsdale, AZ) to generate the top layer. The spacer frame is generated in an analogous manner using a thick (1.5 mm) PMMA sheet laminated on both sides with a pressure-sensitive adhesive (468MP, 3 M, Maplewood, MN). Components were rinsed with isopropyl alcohol and deionized water, loaded with reagents and assembled via lamination prior to use.

### Cartridge reagent preparation and loading

Prior to lamination of the cartridge, the PMMA surface of the wells was passivated with incubation in silicone oil at room temperature for >1 hr. The passivating silicone oil was aspirated immediately before loading the aqueous reagents into each well. The largest well opposite the loading port was loaded with 30 µl binding buffer, the two central wells were loaded with 15 µl wash buffer, and the final well was loaded with 10 µl of the PCR buffer. Single well cartridges designed for directly spiked calibration of PCR contained just the PCR well in the same dimensions as the full cartridge. Magnetic particles, binding buffer, and wash buffer solutions were obtained from a magnetic DNA purification kit (Chargeswitch^TM^ gDNA Mini Bacteria Kit, ThermoFisher Scientific, Walton, MA). A commercial qRT-PCR master mix was used according to the concentrations recommended by the manufacturer for amplification and detection of RNA samples (SensiFast SYBR No-ROX kit, Bioline USA, Taunton, MA). Tween 20 was added as a surfactant to all pre-loaded aqueous reagents for a final concentration of 0.2% to reduce surface tension between the silicone oil and reagent interface for facile magnetic particle transport. After loading the reagents, all cartridge components were laminated and the remaining space within the cartridges was filled with silicone oil (100 cSt, Millipore Sigma, USA). Assembled cartridges were kept on ice before sample injection and processing on our instrument.

### Cartridge temperature control characterization and calibration

A standalone thermistor temperature probe was built using the same design as the probe utilized in the thermal control module of the DM-PCR instrument. This second probe was embedded directly in the PCR well of a calibration cartridge containing 10 µl of water as a surrogate for PCR solution with the remainder of the cartridge filled with silicone oil. The temperature of the heating module was set and held at four different temperatures between 50–105 °C for 30 seconds to obtain a steady state temperature within the PCR well. The linear relationship (R^2^ > 0.99) between the cartridge temperature and heating module was used to calibrate temperature conversions from the desired cartridge temperatures programmed in the graphic user interface to the PID set heating module target temperatures. The same setup with embedded cartridge temperature probe was used to evaluate the accuracy of the calibration for the PCR solution temperature during both thermal cycling (45 cycles, 65–95 °C) and melt (50–95 °C at ~0.3 °C/s) as shown in Fig. 3.

To ensure the sampling of the embedded probe accurately represented the entirety of the PCR solution during thermal cycling, the heat transfer from the aluminum heat block through the cartridge into the PCR solution was simulated using a 3-dimensional finite element model (COMSOL Multiphysics, Burlington, MA, USA). A 2-dimensional cross-section of the center of the heat block and cartridge assembly is shown in Fig. 4. Temperature data from the heat block probe during thermal cycling over three cycles on our instrument was fed into the simulation to set the temperature of the heat block *in silico*. Temperature of the solution, heating block, and cartridge was initialized for the simulation at 95 °C to represent the end state of the hot-start 2-minute hold at 95 °C prior to cycling. The resulting maximum, average, and minimum temperature of the 10 µl aqueous PCR solution modeled within the well from the simulation was compared to results of the thermal cycling temperature measured by the embedded probe (Fig. 4b) with sufficient agreement to conclude the PCR solution achieved uniform heating and cooling for consistent assay conditions.

### Automated magnetofluidic sample purification routine

Sample purification was implemented through pre-programmed motion of opposing permanent magnets around the cartridge by actuation of the Z-θ manipulator. By lowering the top magnet (magnet^T^) onto the top surface of the cartridge by extending the linear servo arm of the Z-θ manipulator, the magnetic particles were concentrated into a plug and extracted from the reagent wells onto the silicone oil on the flat inner surface of the cartridge. Subsequent rotation of the rotary servo provided transfer between wells. Retracting the linear servo arm removed magnet^T^ from the cartridge and raised the bottom magnet (magnet^B^) up towards the wells for attracting the magnetic particle plug into the reagent wells. The low-profile aluminum heat block covering the PCR well was designed thin enough to allow close proximity of magnet^B^ with minimal attenuation of the magnetic field.

The routine for sample purification was as follows. After injecting a sample containing nucleic acids mixed with magnetic particles and binding buffer into a cartridge, magnet^B^ was raised to condense the magnetic particles into a tight plug. Next, magnet^T^ was actuated to the top of the cartridge and rotated above the first wash buffer followed by attraction into the wash buffer with magnet^B^. Washing the magnetic particles of excess serum debris was accomplished by repeated extraction out of the wash buffer with magnet^T^ and attraction back into the well with magnet^B^. This wash process was repeated with transfer into the second wash buffer well. After washing, the particle plug was transferred into the PCR solution and the magnets were moved into a neutral position for 2 minutes to allow for passive elution of the nucleic acids into the solution. The particles were extracted and rotated away from the PCR well prior to NAAT implementation to prevent inhibition of enzymatic activity and allow a clear path for the fluorescence signal.

### p14(ARF) cartridge qPCR and particle transfer evaluation

The ability to conduct a quantitative PCR (qPCR) assay with our cartridge platform was confirmed by directly spiking serial dilutions of synthetic p14(ARF) oligonucleotide targets into PCR solutions with previously validated primers^45^. The PCR solution contained 50 mM Tris-Cl pH 9.2, 16 mM amonium sulfate, 0.05% Brij 58, 3.5 mM magnesium chloride, 0.3 µM of each forward and reverse primer, 200 µM of each deoxynucleotide triphosphate (dNTP), 1 µM EvaGreen dye, and 0.1 µM Klentaq1 DNA polymerase (DNA Polymerase Technology, St. Louis, MO, USA). Target oligonucleotides dilutions with concentrations between 1 nM and 100 aM were spiked into the PCR master mix such that the 10 µl PCR aliquot in each cartridge contained 1 µl of the target solution. Cycling conditions on our instrument were 95 °C for 2 min followed by 45 cycles of 95 °C for 5 seconds and 62 °C for 20 seconds followed by melt from 50 °C to 95 °C at ~3 °C/second. The annealing temperature was chosen given the optimized conditions of the past study using this primer set and cycling was halted at 40 cycles for input target concentrations greater than 10 pM due to early saturation of fluorescence.

### HCV primer design and qRT-PCR

Consensus sequence was identified by aligning the 5′ untranslated region (5′UTR) sequences from reference genomes of all known HCV genotypes obtained from an online HCV sequence database (Los Alamos National Laboratory, Los Alamos, NM). Several candidate primers were subsequently designed based on melting temperature (T_m_). After checking for specificity using Primer-BLAST (National Center for Biotechnology Information, Bethesda, MD) and self-complementarity using Multiple Primer Analyzer (ThermoFisher Scientific, Waltham, MA), the primers were evaluated via qRT-PCR using synthetic HCV 5′UTR RNA standards (Asuragen, Austin, TX). The primer set chosen for implementation on the cartridge was verified on a benchtop instrument for robust amplification of samples spiked with both the synthetic HCV standard (genotype 2b) and all HCV-positive serum samples (genotypes 1a, 2b, 3a). Non-specific amplicon products were typically not present after PCR, or amplified in very late stages of thermal cycling with a distinct melt temperature from the specific product.

### Clinical sample testing

Archived and deidentified serum samples were obtained from the Johns Hopkins School of Medicine and stored at −80 **°**C when not in use. All experimental protocols were approved by the Johns Hopkins Medicine Institutional Review Board, and all methods were carried out in accordance with relevant guidelines and regulations. Informed consent was obtained from all subjects. Serum samples represent two groups of individuals with a history of HCV infection, with the first group having an ongoing HCV infection with a NAAT-detectable viral load, and the second group having been cleared of HCV with no detectable level of HCV RNA. The group with detectable HCV RNA was also tagged with a quantified viral load obtained from a conventional NAAT assay. To conduct a cartridge assay, 10 µl of blood serum at varying dilutions with water was pipette mixed with 4 µl aqueous magnetic particle solution (25 mg/ml in 10 mM MES, 10 mM NaCl, 0.1% Tween 20) and 0.5 µl of carrier RNA (Ambion® Carrier RNA, ThermoFisher, USA) before injection into the binding buffer through the cartridge port followed by mounting the cartridge within our device. Assay protocol on the DM-PCR instrument consisted of four consecutive steps: 1) droplet magnetofluidic processing, followed by 2) 45 **°**C incubation for 10 minutes, followed by 3) 45 cycles of thermal cycling (95 **°**C denature for 5 seconds, 65 **°**C annealing for 20 seconds) and 4) melt curve analysis (positive thermal ramp from 60 **°**C to 95 **°**C at a rate of 0.1 **°**C/s).

## Electronic supplementary material

Supplementary Information
