# Supplementary material for: Sample-to-Answer Droplet Magnetofluidic Platform for Point-of-Care Hepatitis C Viral Load Quantitation
Source: Sci Rep. 2018 Jun 28;8:9793. doi: 10.1038/s41598-018-28124-3 (PMC6023859; doi:10.1038/s41598-018-28124-3)
Supplement: Supplementary file 1 — Supplementary Information [file 41598_2018_28124_MOESM1_ESM.pdf]

Title: Sample-to-Answer Droplet Magnetofluidic Platform for Point-of-Care Hepatitis C Viral Load Quantitation

Authors: Dong Jin Shin[†1], Alexander Y. Trick[†2], Yu-Hsiang Hsieh[3], David L. Thomas[4,5] and Tza-Huei Wang[*1,2,6]

Affiliations:

[1] Department of Mechanical Engineering, Whiting School of Engineering, The Johns Hopkins University, Baltimore, MD, United States

[2] Department of Biomedical Engineering, Whiting School of Engineering, The Johns Hopkins University, Baltimore, MD, United States

[3] Department of Emergency Medicine, School of Medicine, The Johns Hopkins University, Baltimore, MD, United States

[4] Department of Medicine, School of Medicine, The Johns Hopkins University, Baltimore, MD, United States

[5] Infectious Disease Center for Viral Hepatitis, School of Medicine, The Johns Hopkins University, Baltimore, MD, United States

[6] Institute for NanoBioTechnology, The Johns Hopkins University, Baltimore, MD, United States

[†] These authors contributed equally to this manuscript.

[*] To whom correspondence should be addressed: Professor Tza-Huei Wang; Present address: 118B Clark Hall, 3400 N Charles Street, Baltimore, MD 21218, USA; Phone: +1-410-516-7086; E-mail: thwang@jhu.edu

Supplementary Materials

Fig. S1. Fluorescence detection calibration

Fig. S2. Graphic user interface for instrument control

Fig. S3. Magnetic particle transfer

Fig. S4. Cartridge heating calibration

Fig. S5. Analytical evaluation of p14(ARF) PCR assay

Fig. S6. Analytical evaluation of HCV RT-PCR assay

Fig. S7. HCV RT-PCR assay evaluation with clinical samples

Fig. S8. Data processing for DM-PCR cycle threshold and melt analysis

Fig. S9. Cartridge design and fabrication workflow

Table S1. Primer sequences

Table S2. Benchtop HCV-positive PCR data

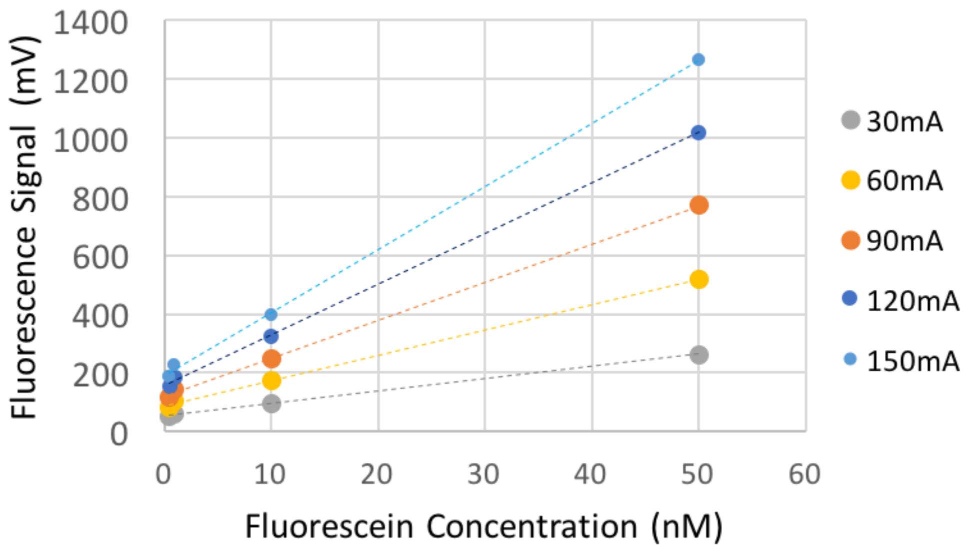

Fig. S1. Fluorescence detection calibration. Cartridges were loaded with 10 µL fluorescein in the PCR well with concentrations of 0.5, 1, 10, 50, and 100 nM, filled with silicone oil, and mounted within our DM-PCR instrument. At 100 nM fluorescein, the detector signal overflowed for all LED excitation settings. The varying LED current settings of the fluorescence detector (Fluo Sens Integrated, QIAGEN, USA) are denoted in the legend. The default LED current of 96 mA was maintained for PCR assays to ensure a strong signal response over large changes in fluorescence without detector overflow.

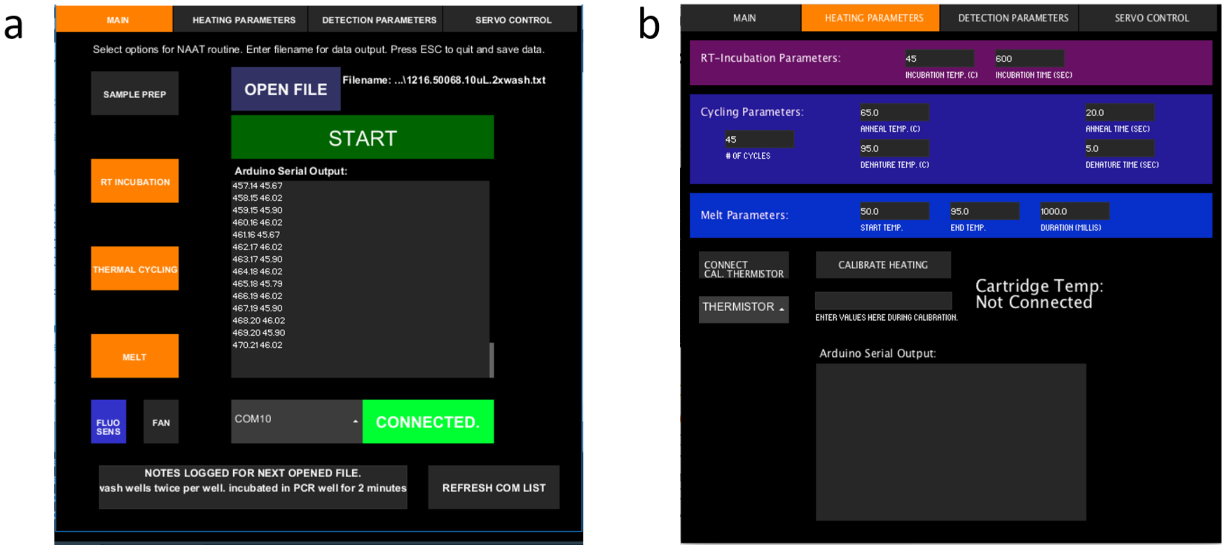

Fig. S2. Graphic user interface for instrument control. (a), The main screen allows the user to selectively turn on magnet sample purification, incubation for reverse-transcriptase steps, thermal cycling for PCR, and melt for analysis. All results are output to text files for automated data analysis using a custom Matlab script. (b), All parameters for temperature control are customizable and temperature calibration with a second thermistor inside a cartridge may be carried out through this screen. Settings for fluorescence detection and calibration of the servos are controlled on subsequent tabs (not shown here).

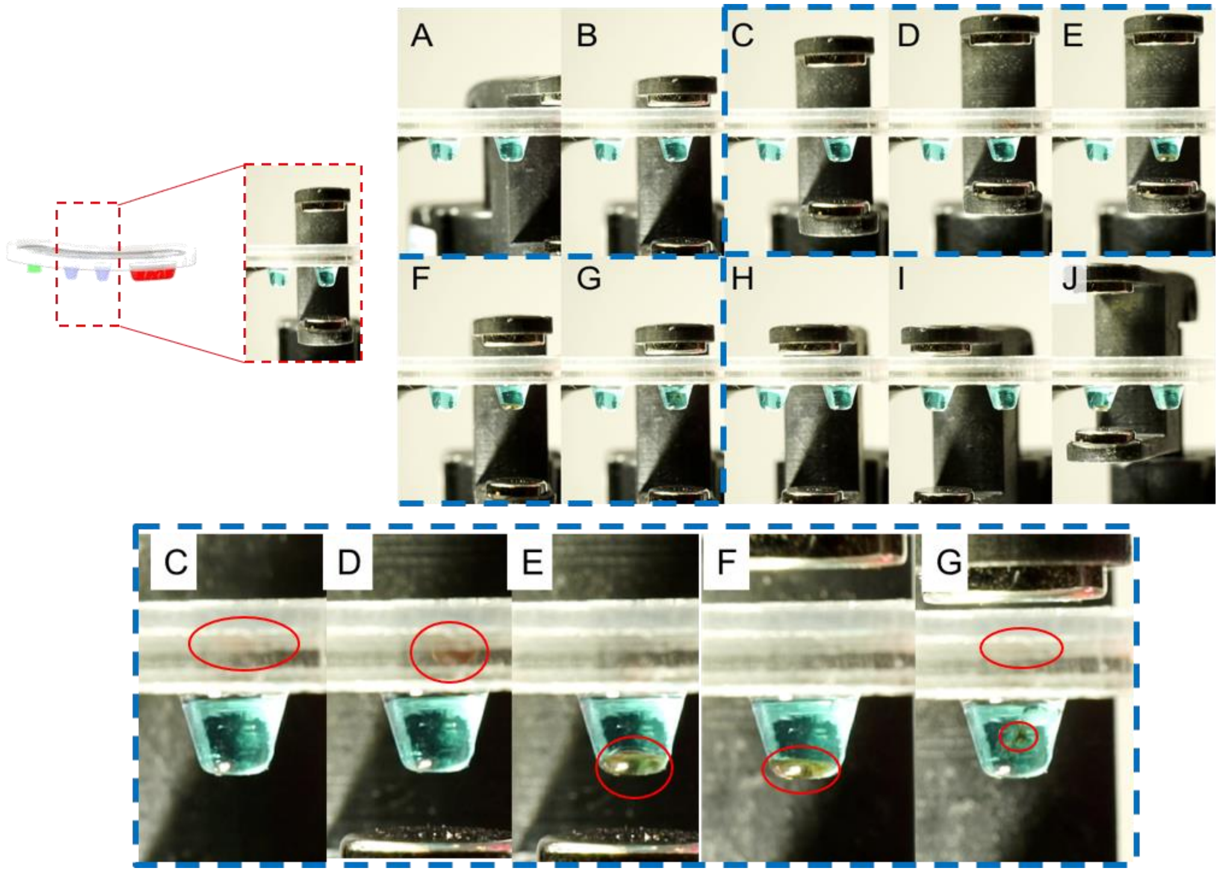

Fig. S3. Magnetic particle transfer. Panels A through J are chronological stills from a video of automated magnetic particle transfer from the binding buffer well into the first wash buffer well and the following transfer into the second wash buffer well. Close-ups of transfer into and out of the first wash buffer are provided for panels C through G. (A) Top magnet (magnet^{T}) has captured particles from binding buffer well and is rotating over the first wash well. (B) Particles are held against top PTFE coating with magnet^{T} positioned above first wash well. (C-E) Raising the bottom magnet (magnet^{B}) draws particles (outlined in red ovals) off the top surface into wash buffer. Panel E close-up shows the particle plug immediately before surface tension with the aqueous buffer squeezes the silicone oil off the particles as evident by the slightly domed appearance. (F) Particles have flattened compared to panel E, indicating silicone oil coating has been squeezed off by the wash buffer. Magnet^{T} has been lowered back down onto the top surface of the cartridge. (G) Particles are attracted to magnet^{T} and coalesce at the PTFE surface. (H-J) Repeat A-D to transfer particles into second wash buffer.

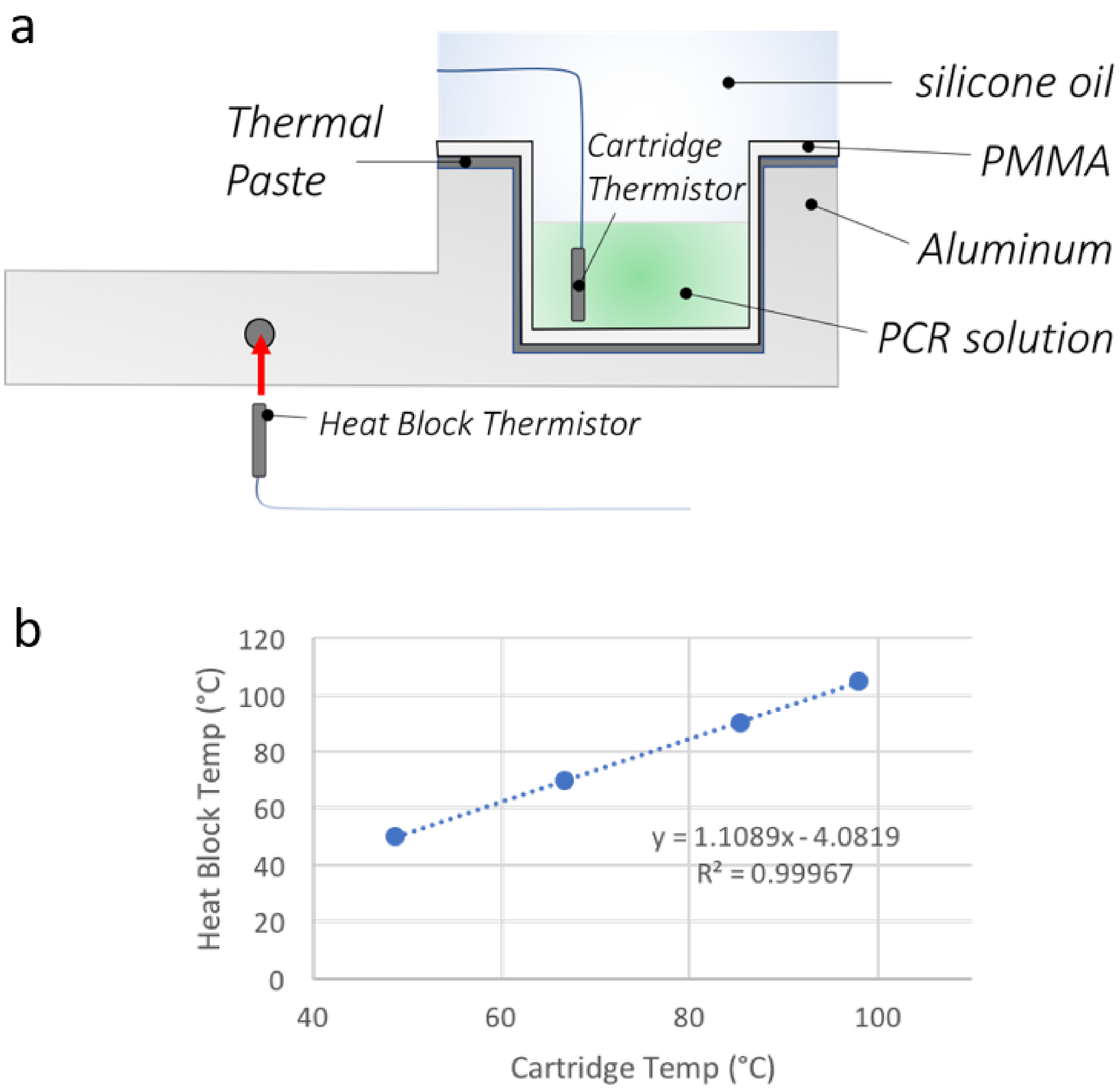

Fig. S4. Cartridge heating calibration. Temperature control for the PCR solution was calibrated using two thermistor temperature probes. The y-axis denotes temperatures detected by the probe embedded in the aluminum heat block, while the x-axis denotes the temperature detected by a second probe directly embedded inside the PCR well of the cartridge. Measurements were recorded after 30 seconds of maintaining a steady-state temperature for the heat block.

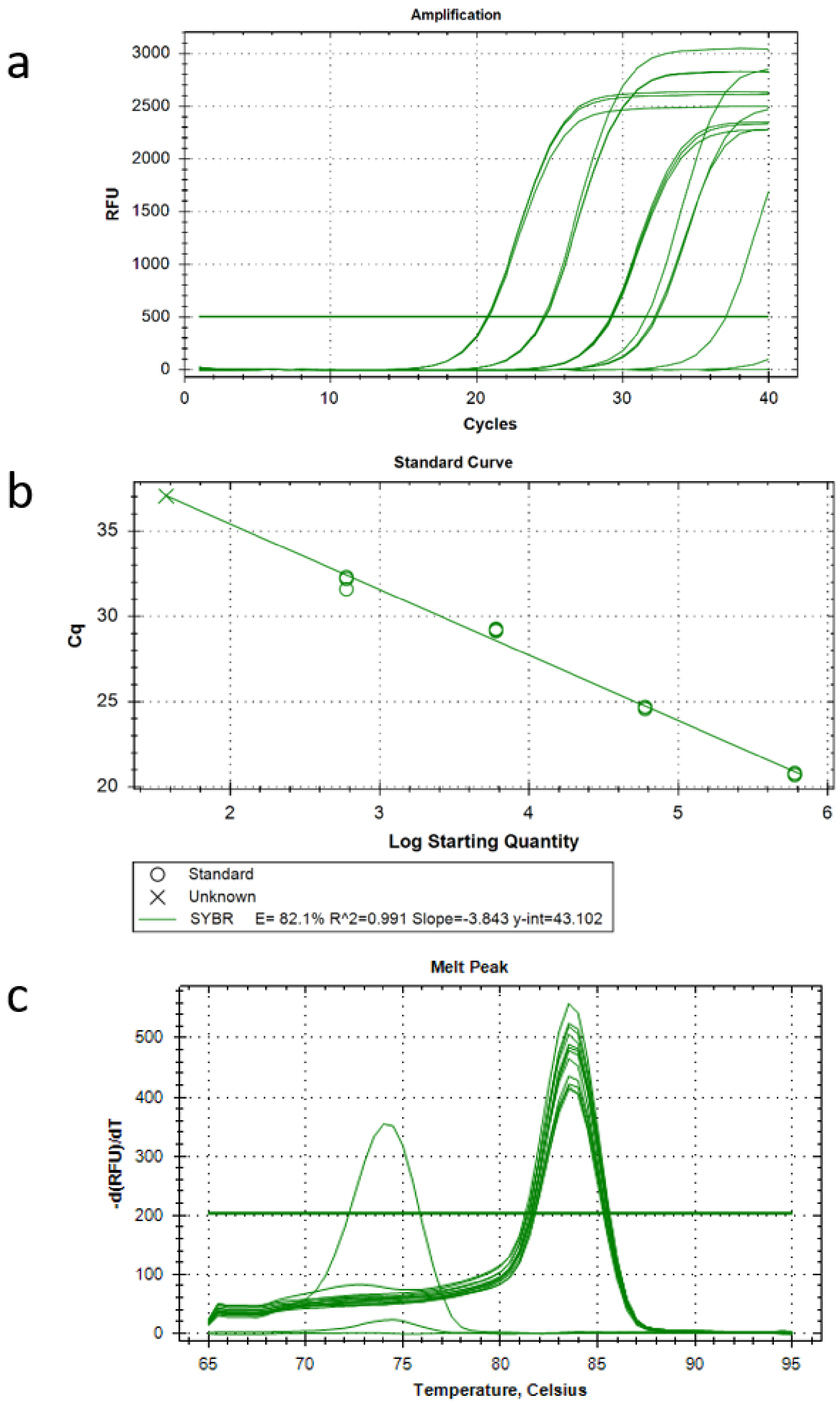

Fig. S5. Analytical evaluation of p14(ARF) PCR assay. (a), real-time PCR curves for 10-fold serial dilutions from 1 pM to 0.001 pM of input p14(ARF) target oligonucleotides including a no-template negative control. All concentrations were tested in triplicate. (b), Standard curve based on threshold cycle number (Cq) from (a). (c), Melt curve analysis shows specific products (Tm = 83.5°C) for all positive samples, while only one of the negative controls amplified with a non-specific product (Tm=74 °C).

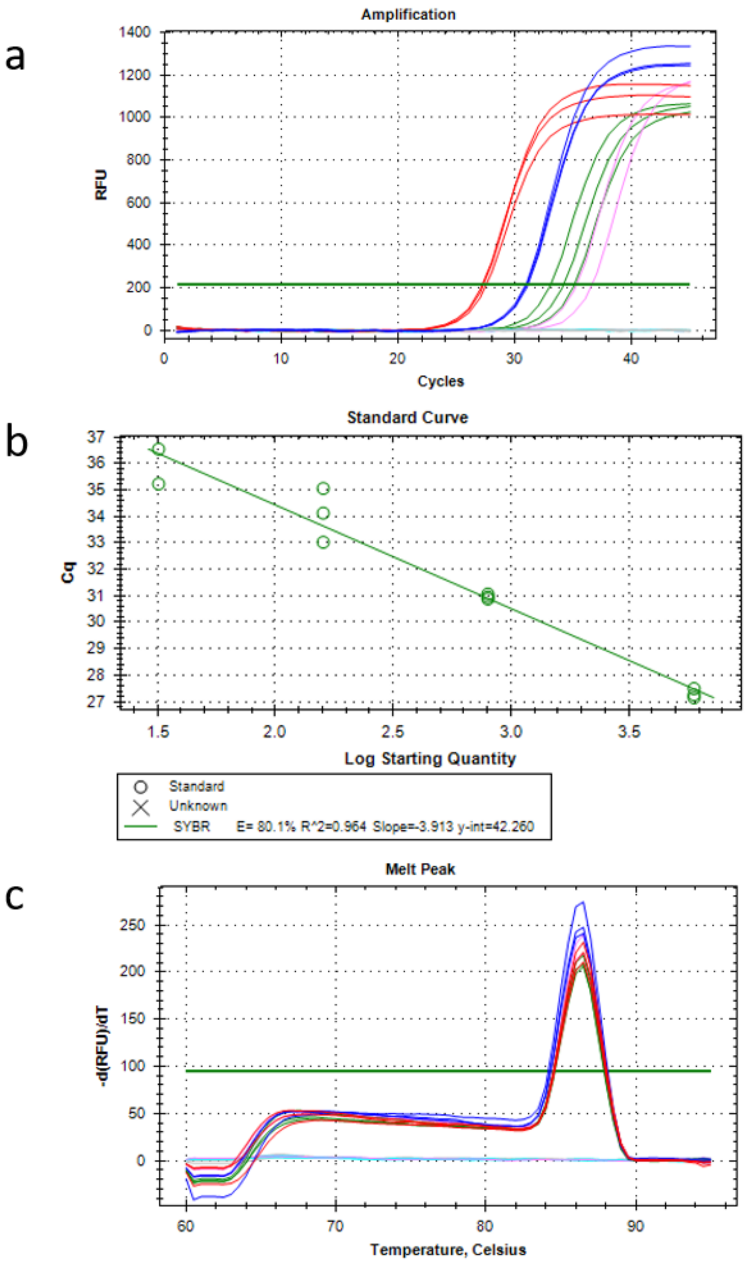

Fig. S6. Analytical evaluation of HCV RT-PCR assay. (a), real-time reverse-transcription PCR curves for 5-fold serial dilution of magnetic-particle purified HCV RNA from patient 1 serum (genotype 1a) corresponding to approximately 6000, 1200, 240, 48, and 9.6 IU HCV RNA per reaction in triplicate. (b), Standard curve based on threshold cycle number (Cq) from (a). Based on the curve, viral load can be quantified in the range of $10^{1.5}$-$10^{3.8}$ IU per reaction (R^2 = 0.96). (c), Melt curve analysis shows specificity of the amplified PCR product to the target RNA template (Tm = 86.5°C) for all positive reactions.

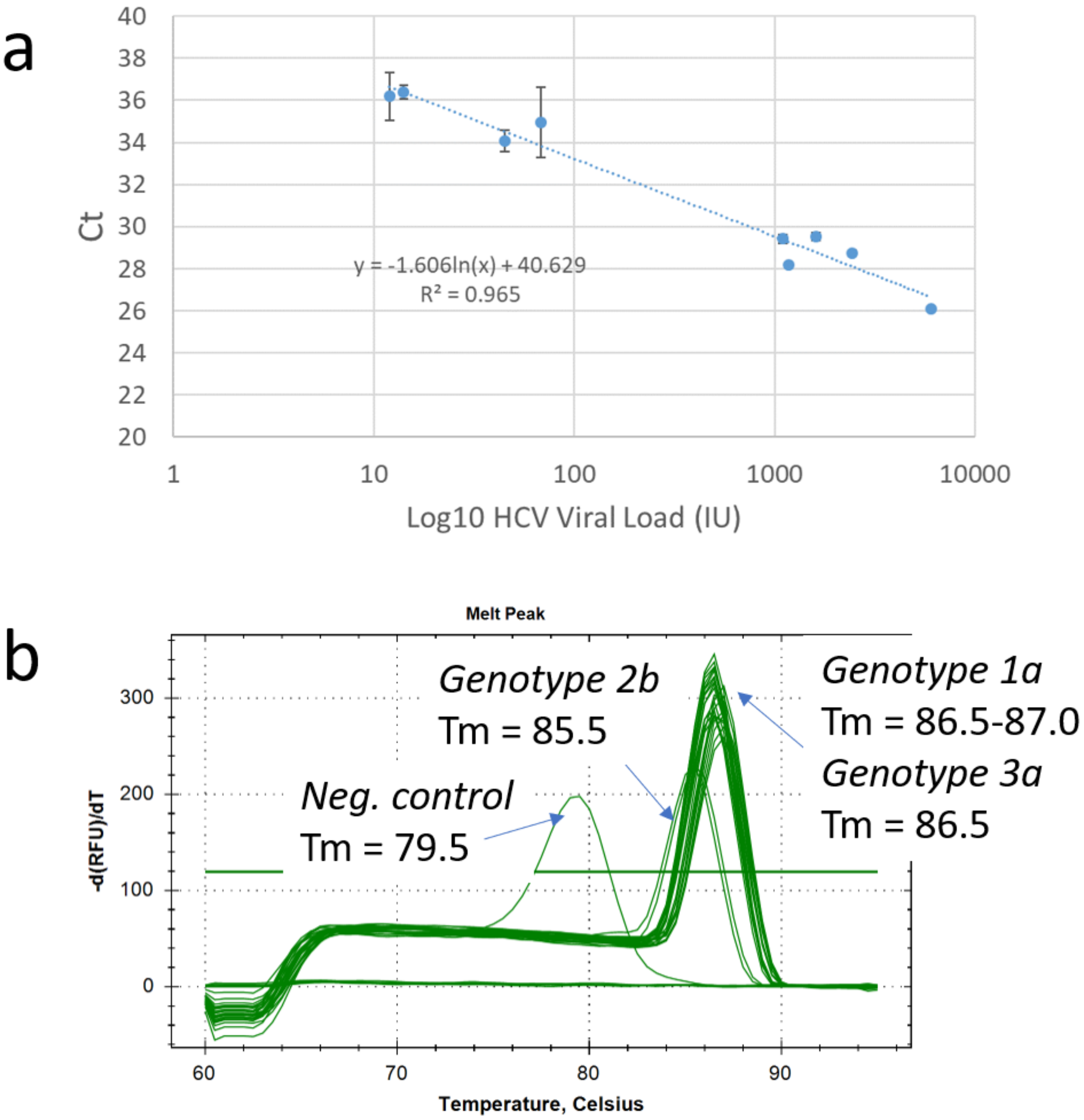

Fig. S7. HCV RT-PCR assay evaluation of all clinical samples. (a), Standard curve generated using IU values as reported by the clinical laboratory versus cycle threshold values from benchtop purification and thermocycling of 1 μL serum per reaction volume. (b), Melt curve analysis of PCR products.

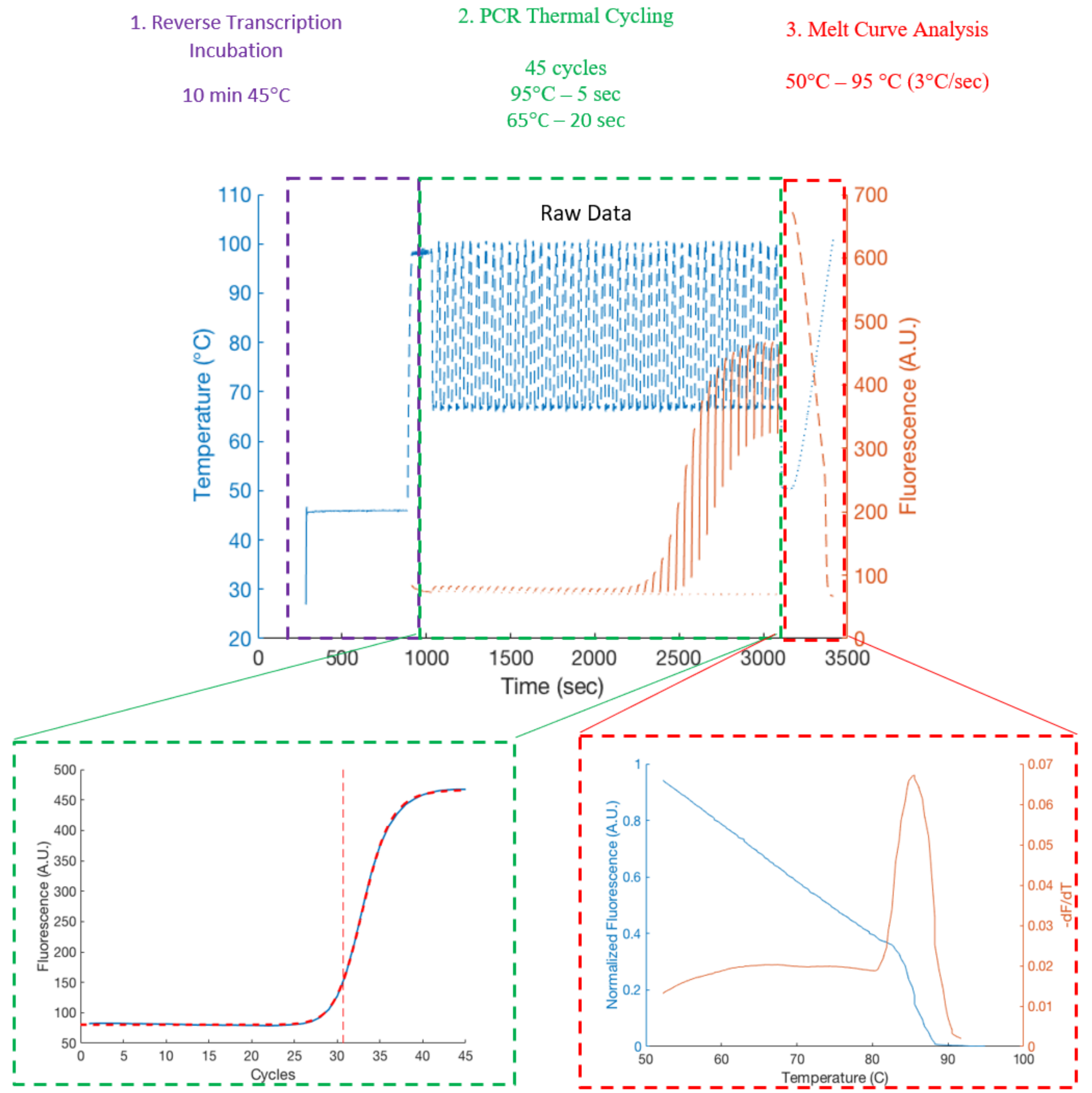

Cycle threshold (Ct) calculation

1. Fluorescence peak extracted from each cycle
2. Logistic regression curve fit (red dashed curvy line)
3. Ct determined by second derivative maximum of curve fit (vertical dashed line)

Melt temperature (Tm) calculation

1. Melt fluorescence curve smoothed with savitzky-golay filter and normalized (blue line)
2. Negative first-order derivative (-dF/dT) calculated with respect to temperature for melt curve (orange line)
3. Tm determined by peak of -dF/dT

Fig. S8. Data processing for DM-PCR cycle threshold and melt analysis

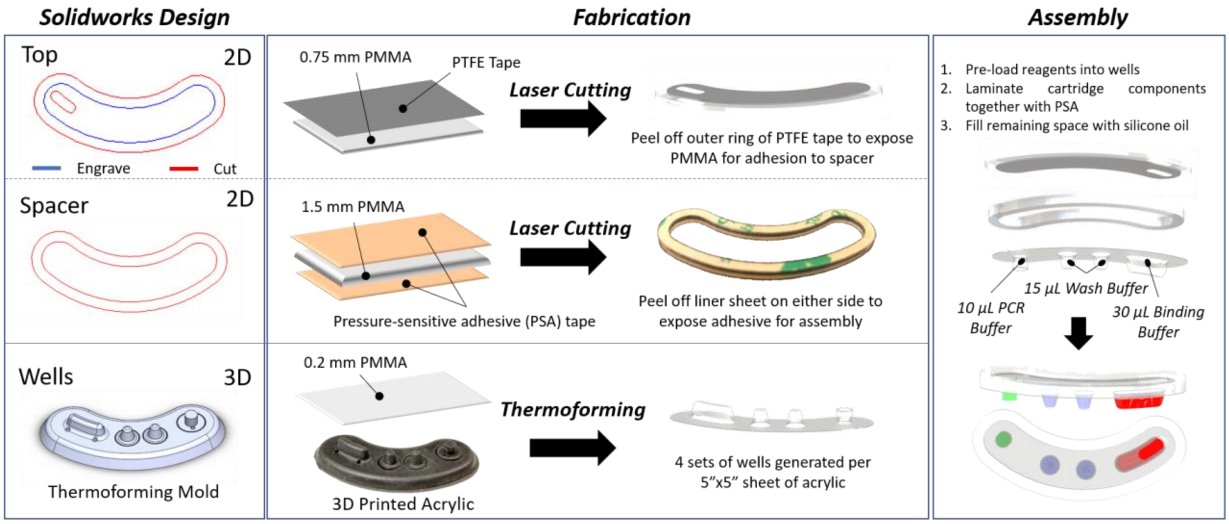

Fig. S9. Cartridge design and fabrication workflow.

Table S1. Primer Sequences

Primer	Sequence	Number of Bases
p14(ARF) FWD	CGTTTTTGGCGTTGTTTATTTT	22
p14(ARF) REV	ACATAATACGCAAATTCTTAATAACCCTC	29
p14(ARF) target	CGTTTTTGGCGTTGTTTATTTTTTCGTGAGTCGCGGGATGT GAATTACGAAAATTTTTATTCGCGGCGGGTCGTACGCGCG TCGAATTCGGAGGGTTATTAAGAATTTGCGTATTATGT	119
HCV FWD	GGAGAGCCATAGTGGTCTGCGGAAC	25
HCV REV	CTCGCAAGCACCCTATCAGGCAGTA	25

Table S2. Benchtop PCR with HCV positive clinical serum samples

Patient	Genotype	Viral Load (IU/uL)	Serum Volume (uL)	Ct1	Ct2	Ct3	Tm
1	1a	6010	1	26.07	26.09	26.08	86.5
2	1a	2440	1	28.86	28.77	28.63	86.5
3	1a	1610	1	29.39	29.49	29.73	86.5
4	1a	1170	1	28.12	28.22	28.17	86.5
5	1a	1100	1	29.35	29.64	29.26	87
6	1a	68.2	1	34.46	33.62	36.83	86.5
7	1a	45	1	33.58	34.05	34.58	86.5
8	2b	14.1	1	36.16	36.63	#N/A	85.5
9	1a	12	1	35.34	35.77	37.48	87
10	3a	3.16	1	#N/A	#N/A	#N/A	#N/A
6	1a	68.2	5	34.09	34.98	33.63	86.5
8	2b	14.1	5	35.7	35.78	36.98	85.5
10	3a	3.16	5	37.4	#N/A	#N/A	86.5
